# Flunarizine Induced Parkinsonism in Migraine Group: A Nationwide Population-Based Study

**DOI:** 10.3389/fphar.2019.01495

**Published:** 2019-12-19

**Authors:** Wei Lin, Cheng-Li Lin, Chung Y. Hsu, Cheng-Yu Wei

**Affiliations:** ^1^ Department of Neurology, Chang Bing Show Chwan Memorial Hospital, Changhua County, Taiwan; ^2^ Management Office for Health Data, China Medical University Hospital, Taichung, Taiwan; ^3^ Graduate Institute of Clinical Medical Science, China Medical University, Taichung, Taiwan; ^4^ Department of Exercise and Health Promotion, College of Education, Chinese Culture University, Taipei, Taiwan

**Keywords:** flunarizine, parkinsonism, drug-induced parkinsonism, migraine, population-based

## Abstract

**Background:** Flunarizine (Fz) is a first-line prophylactic medication that is widely used in migraine. However, Fz has been recognized as a potential cause of drug-induced parkinsonism for a long time. However, to our knowledge, there has been no population-based subgroup analyses for Fz-induced parkinsonism (FIP) in migraine patients.

**Methods:** Data were obtained from the Taiwan’s National Health Insurance Research Database. The study comprised 6,470 migraine patients who were divided into two groups, based on their exposure or non-exposure to Fz.

**Results:** During the study period (2000–2012), the incidence rate of parkinsonism was 1.92 and 8.72 per 1,000 person-years in the control and Fz -treated groups, respectively. In the study population, the adjusted hazard ratio was 4.07 (95% confidence interval CI: 2.84–5.85). In 45–64-year old subjects and ≥ 65-year old subjects, the risk of FIP was 3.18 times (95% CI = 1.63–6.20) and 4.89 times (95% CI = 3.09–7.72) more than that in the controls. The Fz-treated subjects with comorbidities also had a higher risk (4.54, 95% CI: 3.14-6.57). An average annual cumulative Fz dose > 445 mg was accompanied by the greatest risk of FIP; Fz use for >60 days is a cut-off point for predicting future FIP.

**Conclusion:** At the population level, this study showed a complete picture of FIP in migraine patients. FIP is associated with older age, history of comorbidities, exposure to high-dose of Fz, and longer duration of exposure to Fz.

## Introduction

Flunarizine (Fz) is a derivative of piperazine and exerts calcium-channel blocking, anti-histaminic, anti-serotoninergic, and anti-dopaminergic properties. Fz is widely used for migraine, vestibular dysfunction, insomnia, and neuroprotection (Holmes et al., 1984; Brucke et al., 1995). For adult migraine, Fz is considered the first-line prophylactic choice as per several guidelines (Pringsheim et al., 2012; Silberstein et al., 2012; Charles, 2017). A meta-analysis estimated that Fz reduces the headache frequency by 0.4 attacks per 4 weeks (Stubberud et al., 2019). Fz also improves the clocking tinnitus in migraine patients (Chen et al., 2019).

Fz has common adverse effects, such as sedation, weight gain, and depression (Charles, 2017). Drug-induced parkinsonism (DIP) is a serious adverse effect that has been reported from 1984 until today (De Melo-Souza, 1984; Chouza et al., 1986; Micheli et al., 1987; Benvenuti et al., 1988; Capella et al., 1988; Moretti and Lucantoni, 1988; Micheli et al., 1989; Brucke et al., 1995; Negrotti and Calzetti, 1997; Fabiani et al., 2004). However, such studies about Fz-induced parkinsonism (FIP) are limited because of the small sample sizes. Recently, four big data-based studies have focused on the issue (Lin et al., 2017; Liang et al., 2018; Jhang et al., 2019; Kim et al., 2019). The first study concluded that Fz obviously increased the FIP risk, especially in aging, diabetic, or stroke patients (Lin et al., 2017). The second research found that Fz was a potential risk factor for FIP in patients with newly diagnosed type 2 diabetes (Liang et al., 2018). The third indicated that FIP disorders were associated with high-dose or longer exposure, older age, essential tremor, and cardiovascular disease (Jhang et al., 2019). The most recent trial has shown that propulsives, antipsychotics, and Fz are significantly associated with an increased risk of FIP, depending on drug exposure duration and the cumulative dose amount (Kim et al., 2019).

Migraine is a primary headache disorder and the seventh leading cause of time spent disabled in the world (GBD 2015 Disease and Injury Incidence and Prevalence Collaborators, 2016). However, to the best of our knowledge, no population-based analyses have been performed for FIP in migraine patients. This study aimed to investigate the risk factors and cumulative daily dose associated with FIP.

## Methods

### Data Source and Study Design

**Figure 1:**
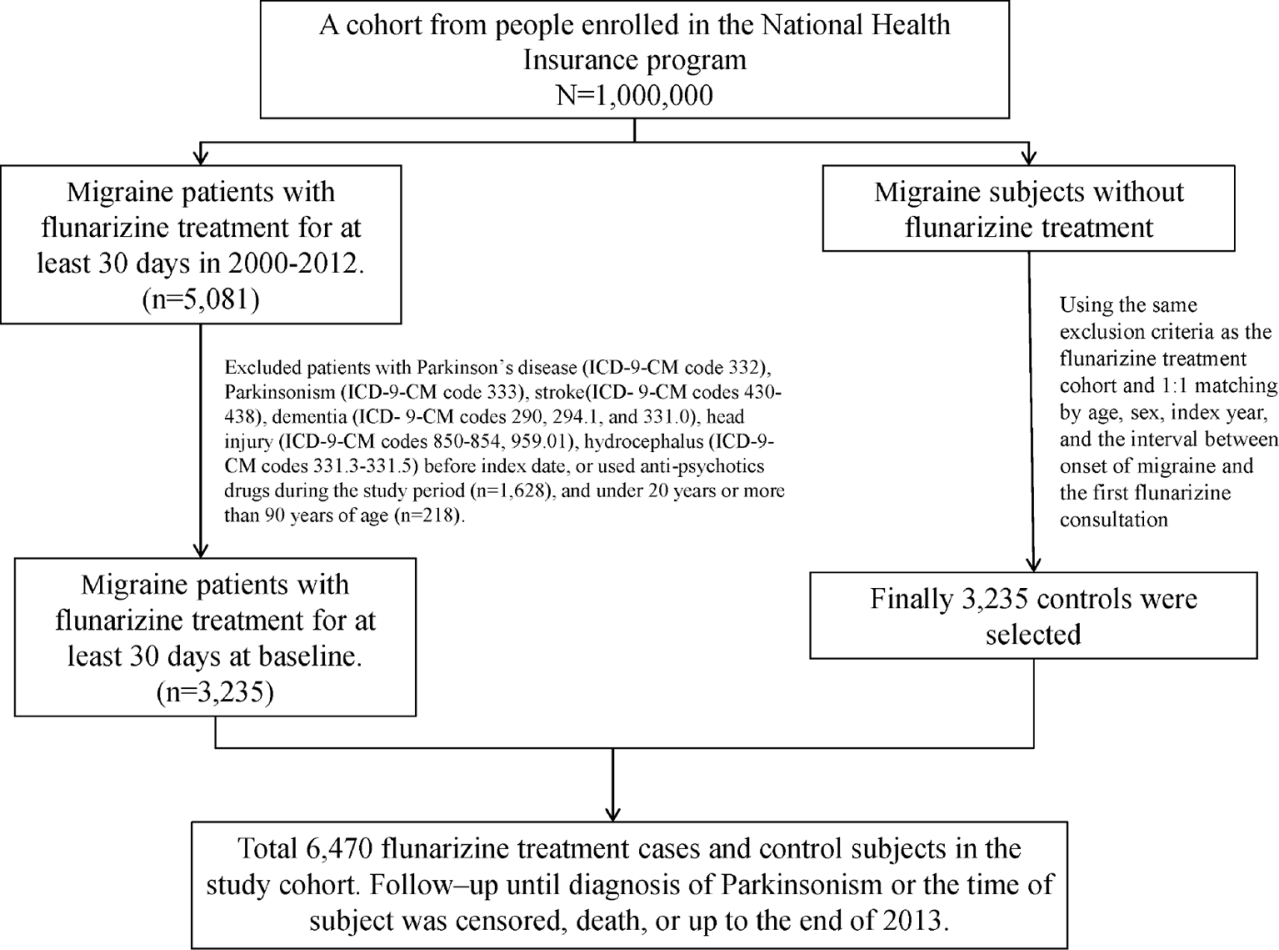
Shows the selection process of the participants in the two study cohorts.

This cohort study utilized the data from the Longitudinal Health Insurance Database (LHID), one of the data subsets of the National Health Insurance Research Database (NHIRD). The NHIRD was established by the National Institutes of Health (NHRI) and has recorded the health information of 99% of the residents in Taiwan. The LHID contained the medical records of one million beneficiaries randomly selected from the National Health Insurance program. The definition of the disease in the LHID was according to the International Classification of Diseases, Ninth Revision, Clinical Modification (ICD-9-CM). The index date was defined as the date of receiving Fz therapy. Each migraine patient (ICD-9-CM 346) who received Fz treatment for more than one month was matched to a subject without Fz treatment by age, sex, index year, and the interval between the onset of migraine and the first Fz consultation. People aged < 20 years or > 90 years were excluded. Moreover, subjects with Parkinson’s disease (PD, ICD-9-CM 332), parkinsonism (ICD-9-CM 333), stroke (ICD-9-CM 430-438), dementia (ICD-9-CM 290, 294.1, and 331.0), head injury (ICD-9-CM 850-854, 959.01), or hydrocephalus (ICD-9 331.3-331.5) and those who used anti-psychotics drugs during the study period were also excluded (**Figure 1**).

### Main Outcome and Covariates

PD or parkinsonism (ICD-9-CM codes 332 and 333, excluding 333.1–333.8) was the main outcome in the present study. The study period was defined as the interval between the index date to the diagnosis of parkinsonism, missing, or death. The PD-related comorbidities, including diabetes (ICD-9-CM 250), hypertension (ICD-9-CM 401-405), hyperlipidemia (ICD-9-CM 272), depression (ICD-9-CM 296.2, 296.3, 300.4, and 311), anxiety (ICD-9-CM 300.00), and sleep disorder (ICD-9-CM 307.4 and 780.5) before the index date were considered covariates.

### Statistical Analyses

Chi-square tests were used to compare the differences in the demographic variables and the comorbidity status between the two cohorts. The mean age in the two groups was examined with a two-sample test. The incidence rates of parkinsonism in difference variables were calculated and the hazard ratios (HR) were also estimated by Cox proportional regression model. We adjusted the HR with the multivariable Cox model that included variables of age; sex; as well as comorbidity of hypertension, diabetes, hyperlipidemia, sleep disorder, anxiety, and depression. A stratified analysis of the duration, average dose, average DDD (defined daily dose), and cumulative DDD (cDDD) of Fz therapy per year was also performed. The Kaplan-Meier method was applied to obtain the cumulative incidence of parkinsonism and compare the difference between the two cohorts with the log-rank test. P-value < 0.05 was considered statistically significant in all the analyses.

## Result

**Figure 2:**
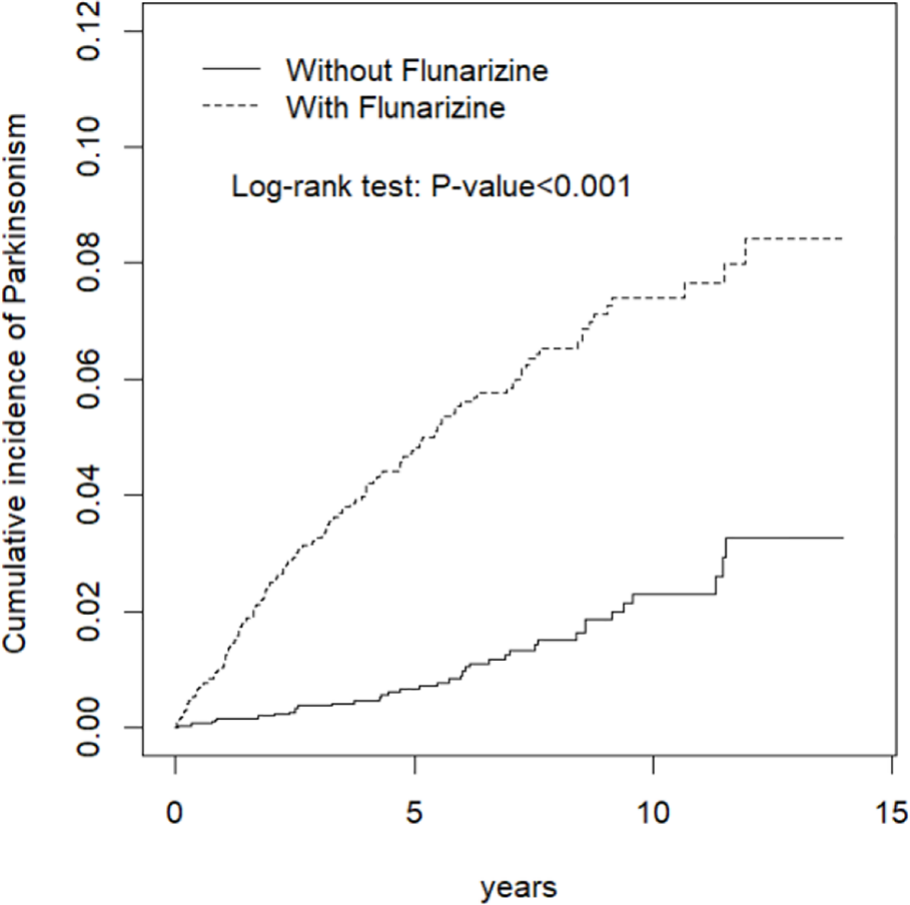
Kaplan-Meir method determined cumulative incidence of parkinsonism compared between Flunarizine cohort and comparisons without Flunarizine.

Total 6,470 subjects were included in this study. The distribution of the demographic and clinical comorbidity status between the two cohorts is shown in **Table 1**. The age and sex in the two cohorts was not significantly different. Most subjects were in the age group of 45–64 years (48.7%), and 74.3% were women. Subjects with Fz treatment had a higher incidence of comorbidities than those without Fz treatment. **Table 2** represents the incidence rate and HR of parkinsonism among the two study cohorts. In those aged 45–64 years and those aged > 65 years, the risk of parkinsonism with Fz treatment was 3.18 times (95% CI = 1.63–6.20) and 4.89 times (95% CI = 3.09–7.72) higher than that in the controls. The adjusted HR of parkinsonism for women in the case group was 4.24-fold (95% CI = 2.69–6.68) higher than that for those in the control group and that for men was 3.89-fold (95% CI = 2.14–7.09) higher. Fz-treated patients with comorbidities had a greater risk of developing parkinsonism (adjusted HR = 4.54, 95% CI = 3.14–6.57). Cox regression analysis stratified by duration and average dose of Fz therapy is displayed in **Table 3**. Patients who received Fz treatment for at least 60 days were more likely to develop parkinsonism than those who had not undergone Fz treatment (adjusted HR = 8.49, 95% CI = 5.86–12.3). The adjusted HR of parkinsonism for patients with Fz dose ≥ 445 mg was 7.69 (95% CI = 5.31–11.1), with Fz average DDD ≥ 45 DDD was 7.77 (95% CI = 5.36-11.3), and with Fz cDDD ≥ 200 cDDD was 4.32 (95% CI = 2.94-6.33). Cumulative incidences of parkinsonism in the two cohorts are shown in **Figure 2**. The curve of the Fz cohort was significantly higher than that of the control cohort, and the p-value of log-rank test was <0.001.

**Table 1:** Distributions of demographic and clinical comorbid status among migraine patients.

	Flunarizine				p-value
	No N = 3235		Yes N = 3235		
	n	%	n	%	
Age, years					0.99
<45	827	25.6	827	25.6	
45-64	1575	48.7	1575	48.7	
≥65	833	25.8	833	25.8	
Mean ± SD^a^	54.1 ± 14.4		54.6 ± 14.3		0.75
Gender					0.99
Women	2405	74.3	2405	74.3	
Men	830	25.7	830	25.7	
Comorbidity					
Hypertension	1360	42.0	1807	55.9	<0.001
Diabetes	220	6.80	313	9.68	<0.001
Hyperlipidemia	1128	34.9	1456	45.0	<0.001
Sleep disorder	1552	48.0	2089	64.6	<0.001
Anxiety	1005	31.1	1769	54.7	<0.001
Depression	461	14.3	901	27.9	<0.001

Chi-square test, ^a^ t-test.

**Table 2:** Incidence and hazard ratio of Parkinsonism for individuals with and without flunarizine among migraine patients.

	Flunarizine						Crude HR (95% CI)	Adjusted HR^a^ (95% CI)
	No			Yes				
	Event	PY	Rate^#^	Event	PY	Rate^#^		
All	38	19780	1.92	166	19030	8.72	4.52(3.18, 6.43)***	4.07(2.84, 5.85)***
Age								
<45	3	5267	0.57	9	5211	1.73	3.05(0.83, 11.3)	1.70(0.44, 6.50)
45-64	12	9703	1.24	44	9502	4.63	3.74(1.98, 7.08)***	3.18(1.63, 6.20)***
≥65	23	4810	4.78	113	4317	26.2	5.39(3.44, 8.44)***	4.89(3.09, 7.72)***
Gender								
Women	24	14596	1.64	108	14172	7.62	4.62(2.97, 7.19)***	4.24(2.69, 6.68)***
Men	14	5184	2.70	58	4858	11.9	4.37(2.44, 7.84)***	3.89(2.14, 7.09)***
Comorbidity^§^								
No	4	5111	0.78	2	1851	1.08	1.42(0.26, 7.75)	1.95(0.35, 10.8)
Yes	34	14669	2.32	164	17179	9.55	4.12(2.85, 5.96)***	4.54(3.14, 6.57)***

CI, confidence interval; HR, hazard ratio; PY, person-years; cHR, crude hazard ratio; aHR, adjusted hazard ratio.

^a^Adjusting for age, gender, comorbidity of hypertension, diabetes, hyperlipidemia, sleep disorder, anxiety, and depression.

^#^Rate, incidence rate per 1000 person-years.

^§^Patients with any one of the comorbidities hypertension, diabetes, hyperlipidemia, sleep disorder, anxiety, and depression as the comorbidity group.

**Table 3:** Incidence and adjusted hazard ratio of parkinsonism stratified by duration, average dose, average defined daily dose, and cumulative defined daily dose of flunarizine therapy per year in migraine patients.

Medication exposed	N	Event	Person-year	Rate	aHR (95% CI)^a^
Fz^#^	3235	38	19780	1.92	1.00
Non- Fz					
<60 days	1660	27	12046	2.24	1.12(0.68, 1.85)
≥60 days	1575	139	6984	19.9	8.49(5.86, 12.3)^***^
<445 mg	1616	27	11617	2.32	1.21(0.73, 1.99)
≥445 mg	1619	139	7413	18.8	7.69(5.31, 11.1)^***^
<45 DDD	1630	28	11715	2.39	1.24(0.76, 2.04)
≥45 DDD	1605	138	7315	18.9	7.77(5.36, 11.3)^***^
<200 cDDD	1661	66	8683	7.60	3.75(2.49, 5.64)^***^
≥200 cDDD	1574	100	10348	9.66	4.32(2.94, 6.33)^***^

CI, confidence interval; Fz, Flunarizine; DDD, defined daily dose; cDDD, cumulative defined daily dose.

^#^The average use day and average dose are partitioned in to 2 segments by median.

^a^Adjusting for age, gender, comorbidity of hypertension, diabetes, hyperlipidemia, sleep disorder, anxiety, and depression.

^*^p < 0.05, ^**^p < 0.01, ^***^p < 0.001.

## Discussion

Fz, with molecular formula C26H26F2N2, is a difluorinated piperazine derivative. It is highly lipophilic, crosses the blood-brain barrier, and is found in higher concentrations in the tissue than in the blood (Leone et al., 1991). Pharmacodynamic studies have shown selective blocking of the entry of calcium into the cells in situations where calcium is stimulated to enter the cell in excess (Holmes et al., 1984). With respect to the possible mechanism of FIP, in addition to the more D2 receptor blocker, loss of tyrosine hydroxylase in the monoaminergic presynaptic neuron may lead to dopamine deletion and cause movement disorders (Negrotti and Calzetti, 1997; Fabiani et al., 2004; Lin et al., 2017).

To our knowledge, this is the first population-based study to investigate the risk of FIP within a 13-year follow-up time in patients with migraine. Our study showed an incidence rate of 8.72% and 1.92% for parkinsonism in the Fz-treated and control groups, respectively. In another study, the incidence rate was 2.9% due to only 3-year follow-up time. For the Fz-treated group, the adjusted HRs were 4.07 and 5.12 (95% CI: 2.84–5.85 and 3.76–6.97) in our migraine subgroup study and another general population-base group, respectively (Lin et al., 2017). There are facts that midlife migraine patients, particularly migraine with aura, have higher risk of PD or parkinsonism in old age more than twice in comparison to people without migraine (Scher et al., 2014). The incidence rate of FIP in the migraine group seems higher then general people. So physicians must look carefully for early signs of PD or parkinsonism in migraine patients treated by Fz.

The higher risk of parkinsonism among the Fz-treated patients was independent of age and comorbidities. The increased incidence rates were 1.73, 4.63, and 26.2 per 1,000 person-years and the adjusted HRs were 1.70, 3.18, and 4.89 (95% CI: 0.44–6.50, 1.63–6.20 and 3.09–7.72) of FIP in young (20–44-year old), middle-aged (45–64-year old), and old (≥65-year old) subjects, respectively. The Fz-treated group with comorbidities had higher incidence (9.55%) and significantly higher adjusted HRs (4.54, 95% CI: 3.14-6.57). Lin et al. reported that elderly, diabetic, or stroke patients had a higher risk. Liang et al. concluded that newly diagnosed type 2 diabetes patients had higher risk of FIP (Lin et al., 2017; Liang et al., 2018). Our results are similar to these previous population-based reports.

Previous studies ever proposed women had higher risk of FIP (Micheli et al., 1987; Teive et al., 2004). Female sex is considered a risk factor for DIP because estrogen can suppress the expression of dopamine receptors (Bedard et al., 1977; Shin and Chung, 2012). But in our study, both male and female patients with Fz treatment showed had a higher tendency to develop parkinsonism compared to the control subjects.

Our study indicates that an average annual Fz cumulative dosage of >445 mg indicates the greatest risk of future parkinsonism. Fz used for >60 days or ≥45 DDD is a cut-off point for predicting future parkinsonism. At the same time, the adjusted HRs changes from 3.75 (95%CI = 2.49–5.64) up to 4.32 (95%CI = 2.94–6.33) in cDDD from <200 to ≥200. Three studies have reported similar results. Liang et al. found that the odds ratio (OR) was 1.77 for patients who used Fz for <1 month, and the OR was up to 7.03 when the exposure period was >3 months. The cumulative dose of Fz also had a linear dose-response effect (Liang et al., 2018). Jhang et al. found that the OR was 3.80 (95% CI: 2.61–5.52) if the cumulative defined daily dose (cDDD) was ≥ 87.75/day. The optimal value of cDDD to predict movement disorders was 58.5 (sensitivity: 0.67, specificity: 0.60), indicating an overall exposure of 585 mg (Jhang et al., 2019). Kim et al. also concluded that Fz had a significant association with increased risk of FIP, depending on the cumulative dose (Kim et al., 2019). Our research provides safety advice of Fz for migraine patients.

Migraine patients are the main users of Fz. Fz is not available in the United States of America but is widely used in Europe as the first-line preventive measure for migraine (Pringsheim et al., 2012; Silberstein et al., 2012; Charles, 2017). One retrospective cohort study by Karsan, N. et al. on 200 migraine patients treated with Fz stated that Fz is generally effective, with only 24% (n = 47) of the patients reporting no clinical effect. The most common dose used was 10 mg per day. Information on treatment duration was available for 39% (n = 78) of the patients. Of these patients, 64% (n = 50) continued treatment for > 1 year. Doses up to 15 mg were generally well tolerated, with only 10.5% (n = 21) of the patients discontinuing treatment because of adverse effects. The most common adverse effects were fatigue (18%), mood change (17%), and weight gain (16%); other less common side effects included tremor (4.5%), dizziness (4%), constipation (2.5%), and nausea (2%) (Karsan et al., 2018). Physicians should carefully weigh the efficacy and adverse effects of Fz when the drug is recommended for long-term use in migraine patients.

This study has certain limitations that should be considered while interpreting the results. First, the NHIRD does not contain detailed information regarding smoking habits, alcohol consumption, socioeconomic status, diet, inactivity, or family history, despite these factors being potential risk factors for parkinsonism. Changes in the lifestyle and diet may affect the results. Second, although the secondary database research lacked clinical information, such as the history, neurological evaluation, clinical course and imaging, some patients may have been wrongly classified. As far as possible to eliminate this limitation, we excluded migraine subjects before Fz treatment with history including PD (ICD-9-CM 332), parkinsonism (ICD-9-CM 333), stroke (ICD-9-CM 430-438), dementia (ICD-9-CM 290, 294.1, and 331.0), head injury (ICD-9-CM 850-854, 959.01), and hydrocephalus (ICD-9 331.3-331.5). After Fz treatment for more than 1 month, we included subjects with PD and parkinsonism. Third, all the data in the NHIRD are anonymous; therefore, relevant clinical variables, such as body mass index, imaging results, and serum laboratory data were unavailable for the study subjects. However, data related to Fz and parkinsonism diagnosis were highly reliable.

In conclusion, Fz is frequently prescribed and is effective for migraine patients. However, FIP is associated with older age, history of comorbidities, a high-dose exposure, and longer exposure duration. Physicians should be aware of the neurogenic adverse effects, especially when the drug is used continuously for >60 days.

## Data Availability Statement

The raw data supporting the conclusions of this article will be made available by the authors, without undue reservation, to any qualified researcher.

## Ethics Statement

The studies involving human participants were reviewed and approved by The Research Ethics Committee (REC) II of China Medical University Hospital. Written informed consent for participation was not required for this study in accordance with the national legislation and the institutional requirements.

## Author Contributions

WL, C-LL, and C-YW participated in the design of the study. C-LL was involved in collecting data and producing tables. WL, C-LL, and C-YW produced the initial draft of the manuscript that was further revised by CH and C-YW. All co-authors reviewed and approved the final version of the manuscript.

## Funding

This work was supported by grants from the Taiwan Ministry of Health and Welfare Clinical Trial Center (MOHW108-TDU-B-212-133004); China Medical University Hospital (CMU106-ASIA-12, CMU107-ASIA-19, DMR-108-207); Academia Sinica Stroke Biosignature Project (BM10701010021); MOST Clinical Trial Consortium for Stroke (MOST 107-2321-B-039 -004-); Tseng-Lien Lin Foundation, Taichung, Taiwan; and Katsuzo and Kiyo Aoshima Memorial Funds, Japan. The funders had no role in the study design, data collection and analysis, decision to publish, or preparation of the manuscript.

## Conflict of Interest

The authors declare that the research was conducted in the absence of any commercial or financial relationships that could be construed as a potential conflict of interest.

## Abbreviations

Fz, Flunarizine; FIP, Fz-induced parkinsonism; DIP, drug-induced parkinsonism; LHID, Longitudinal Health Insurance Database; NHIRD, National Health Insurance Research Database; NHRI, National Institutes of Health; PD, Parkinson’s disease; HR, hazard ratios.
